# Spawning behavior of Aedini (Diptera: Culicidae) in a remnant of Atlantic Forest in the state of Rio de Janeiro

**DOI:** 10.1186/s13071-021-05102-9

**Published:** 2021-11-27

**Authors:** Amanda Queiroz Bastos, Paulo José Leite, Jacenir Reis dos Santos-Mallet, Cecilia Ferreira de Mello, Michele Serdeiro, Júlia Santos dos Silva, Ronaldo Figueiró, Tatiana Docile, Jeronimo Alencar

**Affiliations:** 1 Laboratório de Diptera, Instituto Oswaldo Cruz (FIOCRUZ), Rio de Janeiro, Brasil; 2 Programa de Pós-Graduação em Biologia Animal, Instituto de Biologia, Universidade Federal Rural do Rio de Janeiro, Seropédica, Rio de Janeiro, Brazil; 3 Laboratorio Interdisciplinar de Vigilância Entomológica em Diptera e Hemiptera, Instituto Oswaldo Cruz (FIOCRUZ), Rio de Janeiro, Brasil; 4 Universidade Nova Iguaçu, Nova Iguaçu, Rio de Janeiro, Brazil; 5 Fundação Centro Universitário Estadual da Zona Oeste (UEZO), Rio de Janeiro, Brazil; 6 Centro Universitário de Volta Redonda (UniFOA), Volta Redonda, Rio de Janeiro, Brazil; 7 Brazil Rio de Janeiro,; 8 Laboratório de Educação Profissional em Vigilância em Saúde (LAVSA), Escola Politécnica de Saúde Joaquim Venâncio, Fundação Oswaldo Cruz (FIOCRUZ), Rio de Janeiro, Brazil; 9 Instituto de Aplicação Fernando Rodrigues da Silveira (Cap-UERJ), Universidade do Estado do Rio de Janeiro (UERJ), Rio de Janeiro, Brazil

**Keywords:** Mosquitoes, Oviposition biology, Coexistence, Natural environment

## Abstract

**Background:**

Mosquito assemblages are organized along an ecological gradient, including small habitats where interspecific competition predominates and large permanent habitats where predation predominates. This study aimed to analyze the oviposition behavior of mosquitoes regarding the preference for traps installed at two different heights with regard to ground level and the tendency to share spawning sites in an Atlantic Forest fragment in Nova Iguaçu, State of Rio de Janeiro, Brazil.

**Methods:**

The eggs were collected from April 2018 to March 2019. Twelve ovitraps were used, randomly distributed in trees at ground level and at a height of 3 m in a forest environment.

**Results:**

They were sequentially numbered, monitored, and replaced every 2 weeks. Among the 5818 eggs collected, 3941 hatched, 3756 reached the pupa stage, and 2370 reached the adult stage. The most abundant species were *Aedes albopictus* (63%) and *Haemagogus leucocelaenus* (35%), followed by *Aedes terrens* (2%) and *Haemagogus janthinomys* (1%). Analyses showed a significant difference of (*P* = 0.02) between the number of mosquito species collected in the palettes at ground level and the number collected at the height of 3 m. Cluster analysis of species abundance showed that the eggs collected in the palettes at ground level were more abundant than those collected in the palettes at the height of 3 m. We detected co-occurrence of species in the oviposition palettes; according to the null model, such species distribution was not random.

**Conclusions:**

The exploitation of oviposition sites by mosquito species can represent an event forced by population density facilitated by the ecological valence of individuals of one species. Understanding the aggregate distribution of larvae at the oviposition site allows us to conduct more in-depth studies of the oviposition behavior of female mosquitoes.

**Graphical Abstract:**

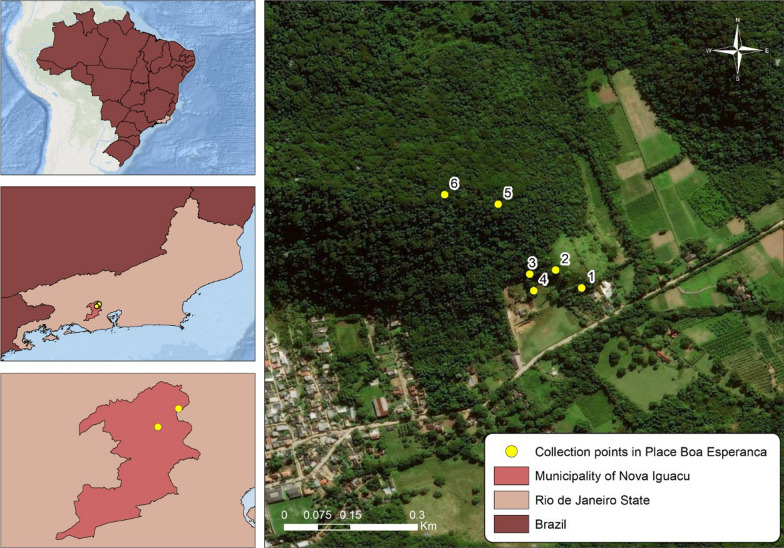

**Supplementary Information:**

The online version contains supplementary material available at 10.1186/s13071-021-05102-9.

## Background

Mosquitoes have attracted considerable attention due to their role in the transmission of important etiological agents such as arboviruses (e.g., Zika, dengue, chikungunya, and yellow fever) and malaria [1], thus leading to severe problems in health services in Brazil. There are 3583 recognized mosquito species, distributed across approximately 113 genera [2], or 42 genera according to the more traditional classification of Wilkerson et al. [3]. The Neotropical region has the highest level of endemicity, with 27% of species restricted to this biogeographic region [4].

According to Mondet et al. [5] and Araújo et al. [6], the yellow fever virus has been detected from north to south of Brazil, with cyclic epizootics, human cases, and virus isolation reported for *Haemagogus janthinomys* (Dyar, 1921) and *Haemagogus leucocelaenus* (Dyar & Shannon, 1924). *Haemagogus janthinomys* is considered the main vector of wild yellow fever (WYF) [7]. However, *Hg. leucocelaenus* is the most common species of the genus in Brazil and was recently found naturally infected with the WYF virus in the state of Rio de Janeiro [7].

The selection of vessels as spawning sites by mosquitoes depends on the species' eclecticism in the use of the type of vessel and the place where it is located. Moreover, some factors can influence the pregnant female’s choice of oviposition site, including color, consistency, and vessel size [8]. Most mosquito species need at least two blood meals for the first egg-laying to develop and for oviposition to occur on the 4th or 5th day [9].

Fader [10] reports a differential resource hypothesis, suggesting that mosquitoes can lay eggs in vessels with high-quality resources. According to Alencar et al. [11], the environmental factors to which the eggs of *Haemagogus* are exposed in the field can influence the hatch rate in the laboratory; therefore, some eggs may need more than one water immersion event to hatch.

Serpa et al. [12] stated that pregnant females of *Aedes aegypti* (Linnaeus, 1762) select vessels containing water with conspecific larval rearing water, suggesting this species’ preference for pre-existing spawning sites in an attempt to reduce the effects of intraspecific competition. Competition for limited resources is much better known than specific differences in resource acquisition and use [10]. The highly asymmetric interspecific competition will often result in the exclusion of the inferior competitor [10].

Mosquitoes from the Aedini tribe show a tendency for temporary spawning sites in wild environments, such as species of the genus *Haemagogus,* which are found colonizing cut bamboos, tree hollows, and coconut shells [13]. Interactions like competition, predation, and mutualism among mosquito larvae can provide valuable models for testing and developing ecological theory for effects dependent on habitat, size, predation intensity, resource availability, or structural complexity [14].

Serpa et al. [12] reported that the effect of water where conspecific and heterospecific larvae hatch as a result of oviposition can contribute to the understanding of the ecological relationships between species as well as assess the reproductive potential of emerging females from spawning sites with coexistence of species.

This study aimed to evaluate the oviposition behavior of mosquitoes regarding the preference for ovitraps installed at two heights in relation to ground level and the tendency to share spawning sites in a fragment of Atlantic Forest in the state of Rio de Janeiro, Brazil.

## Methods

### Study area and sampling

**Fig. 1:**
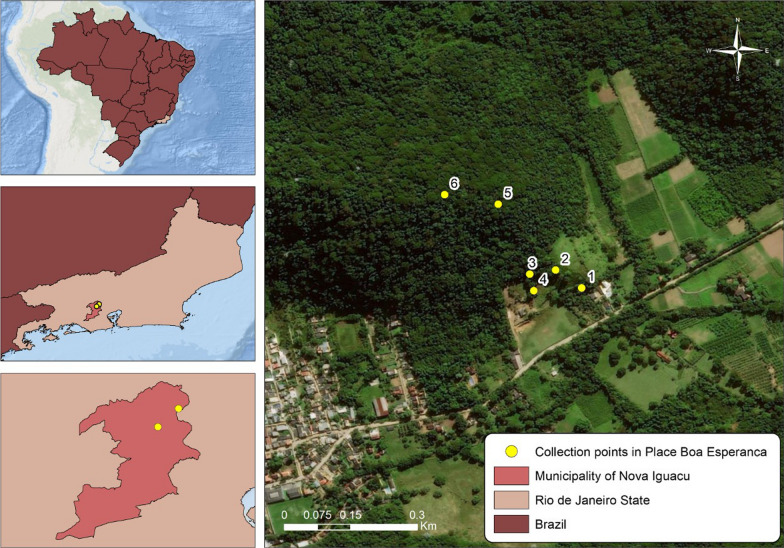
Sampling sites in Boa Esperança farm located in the city of Nova Iguaçu, state of Rio de Janeiro, Brazil. Service layer credits: Source: Esri, Maxar, GeoEye, Earthstar Geographics, CNES/Airbus DS, USDA, USGS, AeroGRID, IGN, and the GIS User Community. On May 4, 2021

Collections were carried out at the Boa Esperança farm (S22°35′16.2''; W043°24′28.9'') located in the city of Nova Iguaçu, state of Rio de Janeiro, Brazil (Fig. 1). The region has a humid tropical climate with an average annual temperature of 21.8 °C and an average annual rainfall of 2105.1 mm [15].

Ovitraps were used for egg collection. This type of trap consisted of a 500-ml lid-less matte black pot with four plywood palettes (Eucatex® plates) held vertically inside the trap by clips. Natural water and litter (remains of leaves and decaying organic material from the soil) were added to the ovitraps to reproduce a microecosystem closer to the natural [4]. Traps were installed at the following collection points under the same conditions, at ground level and at 3 m from ground level.

Collection point 1 (P1S and P1M—S22°35′15.3"; W043°24′29.9") is a secondary forest with recent reforestation and few trees, some nearby dwellings, and livestock. Collection point 2 (P2S and P2M—S22°35′14.0"; W043°24′31.8") is characterized by a clearing with undergrowth and grasses, used for animal grazing. Undergrowth borders the arboreal stratum of collection point 3 (P3S and P3M—S22°35′14.3"; W043°24′33.7"), which is also used for animal grazing. Collection point 4 (P4S and P4M—S22°35′15.3''; W043°24′33.4'') is located around several bamboo plants. Collection point 5 (P5S and P4M—S22°35′09.2"; W043°24′36.0") is situated inside the dense forest, and its vegetation with trees can reach up to 30 m in height. Collection point 6 (P6S and P6M—S22°35′08.5"; W043°24′39.9") has a dense shrub layer with many larger trees and a dense crown. Twelve ovitraps were installed at a random distance, with two traps per tree: one trap in the ground (P1S, P2S, P3S, P4S, P5S, and P6S) and one trap at the height of 3 m (P1M, P2M, P3M, P4M, P5M, and P6M). They were then monitored and replaced every 2 weeks from April 2018 to March 2019. After the palettes were collected, they were placed in a polyethylene box and sent to the laboratory, where they were dried for 2 days at room temperature. Subsequently, the eggs were counted under a stereoscopic microscope.

The palettes were separated in the laboratory, submitted to egg counting, and individually immersed in transparent mesh trays of 27 × 19 × 7 cm containing dechlorinated water.

The eggs were maintained in a controlled experimental environment (greenhouse with thermoperiod and photoperiod), at a temperature of 28° ± 1 °C, relative humidity of 75–90%, and photoperiod of 12 h (day/night). The dwell time of the eggs in the greenhouse was about 3 days, and observations were made daily. After this period, the palettes were removed and the hatched larvae were counted. The palettes were then exposed for drying in the trays and conditioned for 3 days outside the greenhouse for reimmersion.

Species identification was carried out from direct observation of the morphological characters evident under an optical microscope (Leica DMD108®) and consultation of the respective descriptions/diagnoses of the species, using dichotomous keys developed by Lane [16, 17], Arnell [18], Forattini [19], and Marcondes and Alencar [13]. Subsequently, all specimens were deposited into the Entomological Collection of the Instituto Oswaldo Cruz, Fiocruz, Rio de Janeiro, under the title "Atlantic Forest Collection."

### Statistical analysis

To evaluate and compare the diversity of mosquitoes at each collection point, the following indices were used: Shannon-Wiener (H') [20] and Pielou’s evenness index (which measures diversity along with species richness) [21, 22]. Data were tested for normality [23], and those with normal distribution were submitted to a t-test [24] to verify significant differences between specimens collected on the ground and at the height of 3 m. Non-parametric data were tested using the Mann-Whitney U test [25]. These tests were performed in Biostat 5.0 [26, 27]. All statistical tests were performed according to Zar [24].

To evaluate and compare differences in mosquito abundance at different collection points and respective heights, ecological distance was analyzed using cluster analysis, by the unweighted pair group method with arithmetic mean (UPGMA), using the quantitative Bray-Curtis similarity index performed in the statistical program PAST [28]. Analysis of the co-occurrence of spawning species in the same palette was performed in ECOSIM 7.0 [29]; in this analysis, a null model was used to test the hypothesis of observed patterns of species distribution to be random. The C-score index [30] was also calculated to measure the checkerboard units between all possible species pairs. In a community structured by competition, the C-score index should be significantly lower than what would be expected by the null hypothesis. In this analysis, a data matrix is used to calculate average co-occurrence. This matrix generates a series of simulated matrices through a null model to identify whether the calculated index differs significantly from what would be expected by chance.

The following parameters were used in the null model: sums of fixed lines representing the abundance of individuals of the species, in which the total values observed in the lines are kept in the simulation so that the number of occurrences of each species in the null communities is the same of the original data; sums of equiprobable columns, a configuration in which each column (or site) is equally likely to be represented. The values of the co-occurrence indices between each pair of species were also calculated.

## Results

During the sampling period, 1152 palettes were collected from ovitraps that were at ground level and at a height of 3 m. Of this number, 24% (272) were positive for eggs, presenting 5818 eggs, of which 1877 did not hatch. Of the total number of eggs found, 2370 reached adulthood and were identified as *Ae. albopictus* (*n* = 1486; 63%), *Hg. leucocelaenus* (*n* = 823; 35%), *Ae. terrens* (Walker, 1856) (*n* = 46; 2%), and *Hg. janthinomys* (*n* = 15; 1%) (Additional file 1: Table S1). Considering the overall sum, all species found occurred in greater abundance at ground level. *Haemagogus leucocelaenus* was more abundant in the forest area (point 5), showing the transition to collection point 1, where *Ae. albopictus* was more abundant (Additional file 1: Table S1). Compared to *Ae. albopictus*, *Hg. leucocelaenus* was more frequent in traps located at the height of 3 m; however, 79% of the specimens preferred to spawn in the traps installed at ground level.

**Fig. 2:**
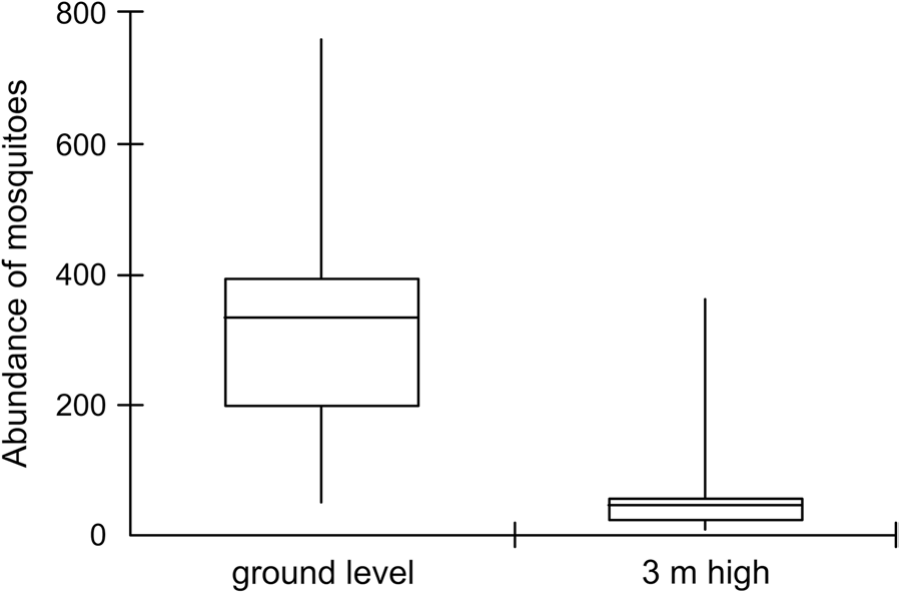
Boxplot expressing the statistically significant result of the Mann-Whitney test for the abundance values of mosquito species in traps at ground height and above 3 m. Mann-Whitney Test *U* = 6.00; *Z* = 1.92; *P* = 0.02*

The Mann-Whitney test (*U* = 6.00; *Z* = 1.92; *P* = 0.02) revealed a significant difference in the abundance of mosquito species found in the palettes collected at ground level compared to those installed at the height of 3 m (Fig. 2). In the palettes collected at ground level, a greater abundance of mosquito species was observed. However, there was no significant difference in the other community parameters calculated (richness, diversity, dominance, and evenness) among the six collection points of the analyzed species (Additional file 2: Table S2). The greatest diversity values were observed in the traps located at ground level, at collection points 3 (*H*' = 0.8367) and 5 (*H*' = 0.8055), whereas the highest richness values were at collection points 5 and 6 (*S* = 4 for both points) (Additional file 2: Table S2).

**Fig. 3:**
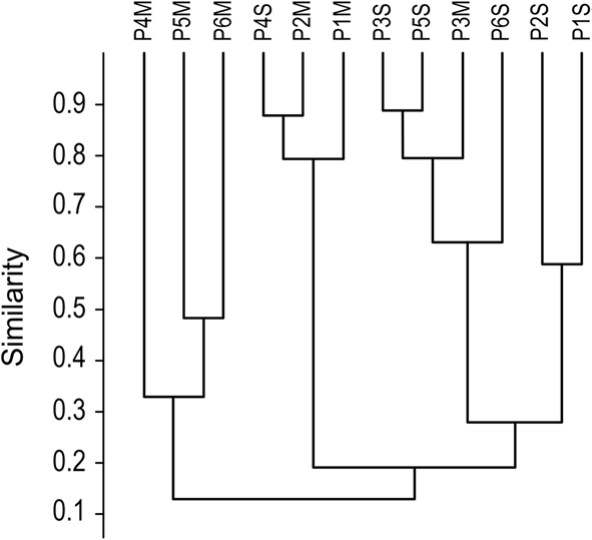
Cluster analysis (UPGMA method) based on quantitative values of mosquito species abundance (Bray-Curtis index = 0.91)

The cluster analysis (Bray-Curtis index = 0.91) applied to species abundance indicated that the palettes collected at ground level had the highest abundance of specimens, comprising a group with the collection points P1S, P2S, P3S, P5S, and P6S, while the palettes collected at the height of 3 m (P1M, P2M, P4M, P5M, and P6M) formed another less abundant group (Fig. 3). Only P4S at the ground level and P3M at the height of 3 m did not fit the expected abundance of these groups (Fig. 3).

**Fig. 4:**
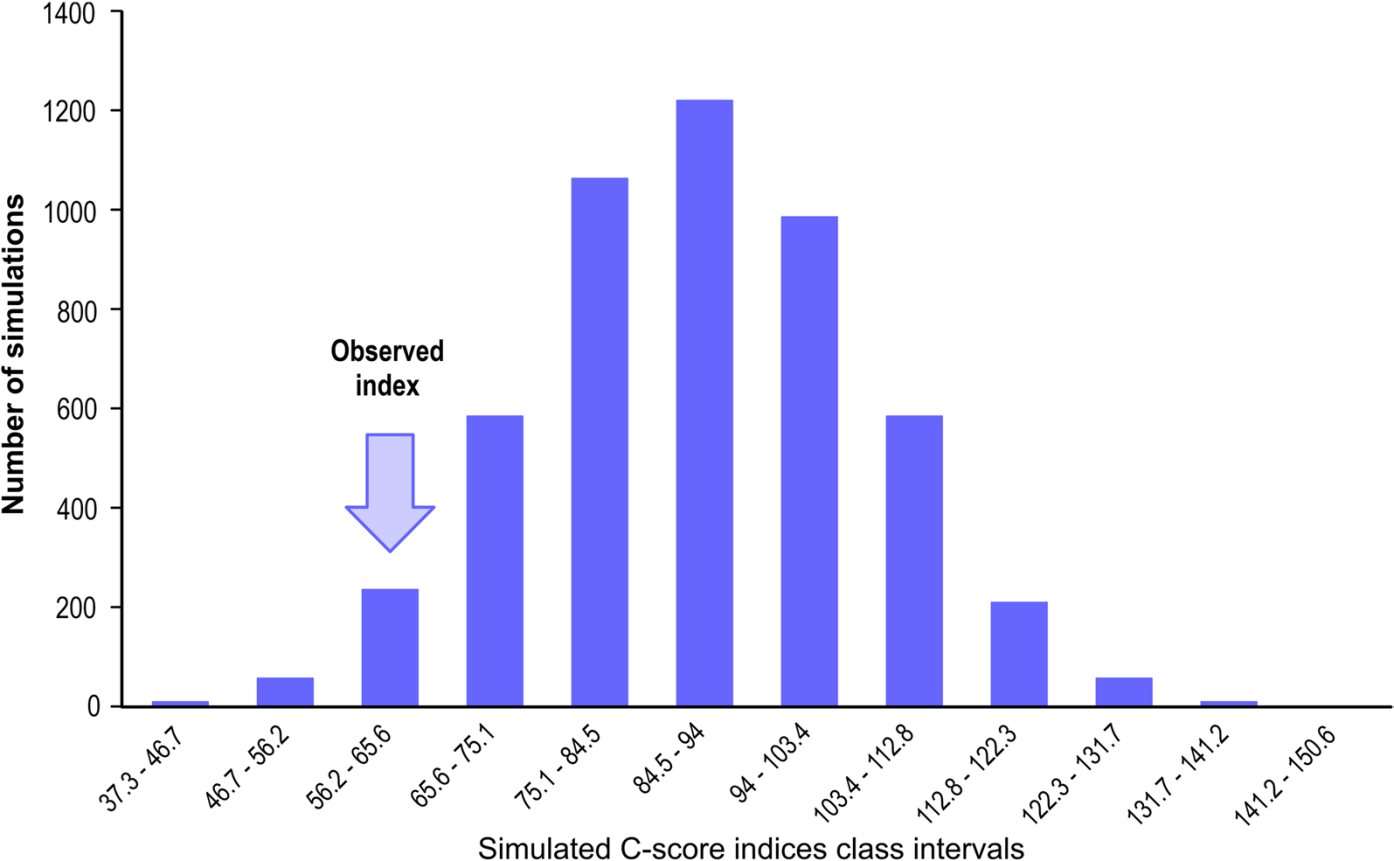
Null model for co-occurrence of immatures [observed index = 60.83, mean of simulated indices = 89.05, variance of simulated indices = 225.80, *p* (observed ≤ expected) = 0.02, *p* (observed ≥ expected) = 0.97]. The arrow indicates the value of the co-occurrence index from actual data

According to the null model, co-occurrence and distribution of all species found in the different collection points were not random (Additional file 3: Table S3). The distribution was structured: observed index = 60.83; mean of simulated indexes = 89.05; variance of simulated indexes = 225.80; *p* (observed ≤ expected) = 0.02; *p* (observed ≥ expected) = 0.97 (Fig. 4). The C-score index was significantly lower than expected by the null hypothesis (Additional file 3: Table S3; Fig. 4).

The graph of this analysis shows arrows pointed to the value of the C-score index observed. This value is d in the part of one of the tails of the normal curve, indicating that it is significantly lower than expected by the null hypothesis (Additional file 3: Table S3; Fig. 4).

## Discussion

The present study suggests an ecological valence in the oviposition behavior of *Ae. albopictus*, which was able to cohabit with other species in the same palette and was successful in surviving compared to other species found during the sampling period. Barbosa and Navarro-Silva [31] reported that *Ae. albopictus* has plasticity in the oviposition behavior, a characteristic related to colonization that is a critical epidemiological factor.

Analysis of the abundance showed that *Ae. albopictus, Hg. leucocelaenus*, and *Ae. terrens* were more abundant in traps located on the ground, while *Hg. janthinomy* was more abundant in traps installed at the height of 3 m. Silva et al. [32] recorded partially differentiated results, as they found the highest abundance at the height of 3 m for the last three species mentioned. In their study, *Ae. albopictus* showed the highest number of specimens at ground level, with 52% of specimens collected.

The reproductive success of mosquitoes is related to selection of the oviposition site, a critical factor for survival and population dynamics. This selection influences larval distribution in the habitat and results from complex interactions of chemical and physical factors [33].

*Aedes albopictus* showed greater dominance during the sampling period. Zequi et al. [34] reported the wide occupation of spawning sites and the ecological valence of *Ae. albopictus* when colonizing wild and anthropic environments, laying posture in both natural and artificial spawning sites.

Honório and Lourenço-de-Oliveira [35] found *Limatus durhamii* Theobald, 1901, coexisting with *Ae. albopictus* and *Ae. aegypti*, suggesting that coexistence and the presence of immature forms of the same species in a spawning site indicate favorable conditions for species development. It is important to emphasize that 38 of the 272 positive palettes were shared by species, with 36 palettes shared by 2 species and 2 palettes shared by 3 species.

Beier et al. [36] observed that, although several species might cohabit, only one or two predominate. Similarly, in palettes with eggs of *Hg. leucocelaenus*, the coexistence of two and three species in the same palette was detected in this study*.* Marques et al. [37] reported eggs from *Ae. terrens* found with those *Ae. albopictus*.

In addition, *Ae. albopictus* shared palettes with *Ae. terrens*, *Hg. leucocelaenus*, and *Hg. janthinomys*. This result differs from those of Alencar et al. [38], who found the eggs of only one species in the palette, and Chadee et al. [39], who reported that females have a tendency not to lay eggs in places where their eggs or those of other species are already found.

Evaluating the oviposition behavior of mosquitoes can contribute to developing strategies to attract pregnant females and monitor the early presence of species in anthropic ecosystems [31].

Analysis of the null model for co-occurrence revealed a smaller number of co-occurrences than expected by the null hypothesis; if co-occurrences were random, the value of the C-score index would be higher. These findings reveal an ecological phenomenon structuring the population distribution, with competition possibly playing a role in the observed patterns, which structured the observed combination of species. Docile et al. [40] reported that immatures collected from bromeliads presented a structured distribution of specimens, not following a random pattern.

Schoener [41] suggested that niche overlapping observed in nature should occur at a lesser degree than expected at random and that a significantly small observed overlap may imply competition and resource partitioning [42]. Thus, the results of our simulations are consistent with the hypothesis that the observed patterns were not random and that the community structure was partially driven by competition and partitioning of a possible limiting resource; in this case, the space available when sharing the palettes inside the ovitraps.

Price [43] argued that female mosquitoes in the laying phase need to find a suitable place for daily oviposition. Females of different species should be expected to prefer oviposition sites associated with favorable conditions for the offspring [43]. Preferences for a particular type of spawning site for oviposition are exhibited by many mosquito species [33, 44, 45], and this choice is the result of a complex interaction between biotic and abiotic factors on which the survival of the aquatic stages of mosquitoes depends.

The search for these factors requires studying the use of the habitat (in this study, the ovitraps) and how this space and its resources are shared by the species found [46]. Therefore, the initial increase in mosquito populations in the spawning site should stabilize in response to reducing the amount of resources. Thus, oviposition behavior can influence the result of competitive interactions between species and, consequently, the structure of the mosquito community in the palettes.

Ecological successions carried out by different species can be responsible for the longest time with high population density due to the colonization of different species at different times of the year [8].

In a study conducted in Nova Iguaçu, Brazil, Honório and Lourenço-de-Oliveira [35] reported that Aedini larvae were rare in the winter period, especially in August, and more frequent in February, coinciding with the period of greatest rainfall. In line with the findings of those authors, the species of Aedini presented the lowest abundance in the winter and the highest frequency in the summer in the present study.

Couto-Lima et al. [47] reported that some Aedini species with desiccation-resistant eggs might die or migrate over a few months during the unfavorable weather season. However, those authors found *Hg. leucocelaenus* throughout their sampling period. On the other hand, no eggs of *Hg. leucocelaenus* were collected during winter in the present study.

Zequi et al. [34] reported that all species collected had a decrease in their population densities in the coldest months of the year. This direct influence of temperature on the activity of mosquitoes, as well as the population increase in the hottest and rainiest periods of the year, is widely known [34].

The eggs of *Ae. terrens* were found exclusively in the ovitraps installed at ground levels. Similarly, Alencar et al. [38] found that *Hg. janthinomys* was predominant in the palettes collected from the tops of the trees and *Ae. terrens* in the traps installed in the ground. In the present study, *Ae. albopictus* was dominant at the two heights where the ovitraps were installed, and *Hg. leucocelaenus* showed greater abundance in the collection point with dense forest.

Tátila-Ferreira et al. [48] suggested that the availability of resources is the leading indicator of the abundance of *Hg. leucocelaenus* in the different strata. Indeed, several authors consider the oviposition site as the main factor affecting the dispersal of female mosquitoes (e.g., Tátila-Ferreira et al. [48]).

Camargo-Neves et al. [49] found that *Hg. leucocelaenus* has a greater ability to adapt to modified environments when compared to other species of the genus. Their findings are in line with the present study, in which *Hg. leucocelaenus* was abundant in the sample site with the same vegetation characteristics.

Fader [10] suggests that mechanisms that alter inter- and intraspecific competition can explain variable coexistence patterns and predict future species distribution as well as understand the consequences for resident species that interact with mosquitoes.

The cluster analysis shows a group of the most abundant species of eggs collected from the traps installed at ground level and another group formed with eggs of less abundant species in the palettes collected at the height of 3 m. It should be noted that this finding is also evident when analyzing the absolute numbers of specimens collected at ground level and at the height of 3 m. Alencar et al. [4] observed that mosquito diversity was higher in samples collected from ovitraps located at ground level and that the frequency decreased according to the height of the trap. This result can be explained by the low supply of hosts for feeding mosquitoes with blood [4].

Studying mosquitoes in a natural environment is essential for assessing possible changes in the behavior and adaptation of these insects according to the environmental conditions of regions that have undergone or are undergoing changes caused by humans, especially for epidemiologically important species [4]. Mosquito populations are organized along an ecological gradient that includes ephemeral habitats where interspecific competition predominates, and large predators are uncommon to large permanent habitats where predation predominates [14].

## Conclusions

Our study suggests a pattern of species distribution related to intra- or heterospecific competition and the height at which females select the oviposition site. We believe that factors such as food resources should be investigated to understand the pattern of the population distribution of species of the Aedini tribe.

## Supplementary Information

**Additional file 1: Table S1.** Absolute abundance of species from the Aedini tribe by collection point (1 to 6) collected on the ground and height 3 m in the sampling period from April 2018 to March 2019 at the Boa Esperança site in Tinguá, Municipality of Nova Iguaçu, State of Rio de Janeiro.

**Additional file 2: Table S2.** Values obtained for richness, abundance, dominance, Shannon (α) diversity, and Pielou’s equitability for each of the 12 studied traps and the respective test values and *p*. (*)*p* < 0.05.

**Additional file 3: Table S3**. Values of the co-occurrence C-score indices between each pair of mosquitoes species found.

## Data Availability

Not applicable.
