# Supplementary material for: Spawning behavior of Aedini (Diptera: Culicidae) in a remnant of Atlantic Forest in the state of Rio de Janeiro
Source: Parasit Vectors. 2021 Nov 27;14:591. doi: 10.1186/s13071-021-05102-9 (PMC8626988; doi:10.1186/s13071-021-05102-9)
Supplement: Supplementary file 1 — Additional file 1: Table S1. Absolute abundance of species from the Aedini tribe by collection point (1 to 6) collected on the ground and height 3 m in the sampling period from April 2018 to March 2019 at the Boa Esperança site in Tinguá, Municipality of Nova Iguaçu, State of Rio de Janeiro. [file 13071_2021_5102_MOESM1_ESM.doc]

**Table S1** Absolute abundance of species from the Aedini tribe, by collection point (1 to 6) collected on the ground and height 3 meters, in the sampling period from April 2018 to March 2019 at the Boa Esperança site in Tinguá, Municipality of Nova Iguaçu, State of Rio de Janeiro.

| Species/Sites | P1 | | P2 | | P3 | | P4 | | P5 | | P6 | | Total | | Total |
| --- | --- | --- | --- | --- | --- | --- | --- | --- | --- | --- | --- | --- | --- | --- | --- |
|  | ground | 3m | ground | 3m | ground | 3m | ground | 3m | ground | 3m | ground | 3m | ground | 3m |  |
| *Ae. (Ste.) albopictus* (Skuse, 1895) | 738 | 54 | 335 | 36 | 120 | 55 | 42 | 1 | 99 | 1 | 4 | 1 | 1338 | 148 | 1486 |
| *Ae. (Pro.) terrens* (Walker, 1856) | 15 | 0 | 1 | 0 | 16 | 0 | 0 | 0 | 13 | 0 | 1 | 0 | 46 | 0 | 46 |
| *Hg. (Hag.) janthinomys* Dyar, 1921 | 0 | 4 | 0 | 3 | 0 | 0 | 0 | 0 | 4 | 2 | 1 | 1 | 5 | 10 | 15 |
| *Hg. (Con.) leucocelaenus* (Dyar & Shannon, 1924) | 7 | 1 | 71 | 4 | 183 | 105 | 6 | 4 | 229 | 47 | 152 | 14 | 648 | 175 | 823 |
| Total | 760 | 59 | 407 | 43 | 319 | 160 | 48 | 5 | 345 | 50 | 158 | 16 | 2037 | 333 | 2370 |
|  | 820 | | 450 | | 495 | | 60 | | 414 | | 181 | | 2370 | |  |

Collection point: (P1, P2, P3, P4, P5 and P6)
