# Supplementary material for: Spawning behavior of Aedini (Diptera: Culicidae) in a remnant of Atlantic Forest in the state of Rio de Janeiro
Source: Parasit Vectors. 2021 Nov 27;14:591. doi: 10.1186/s13071-021-05102-9 (PMC8626988; doi:10.1186/s13071-021-05102-9)
Supplement: Supplementary file 2 — Additional file 2: Table S2. Values obtained for richness, abundance, dominance, Shannon (α) diversity, and Pielou’s equitability for each of the 12 studied traps and the respective test values and p. (*)p < 0.05. [file 13071_2021_5102_MOESM2_ESM.doc]

**Table S2 Values obtained for Richness, Abundance, Dominance, Shannon (α) Diversity and Pielou’s Equitability for each of the twelve studied traps and the respective test values and p. (*) p< 0.05.**

| Sites | Richness | Abundance | Dominance | α diversity | Equitability |
| --- | --- | --- | --- | --- | --- |
| P1S | 3 | 760 | 0.9434 | 0.1492 | 0.1358 |
| P2S | 3 | 407 | 0.7079 | 0.4796 | 0.4366 |
| P3S | 3 | 319 | 0.4731 | 0.8367 | 0.7616 |
| P4S | 2 | 48 | 0.7813 | 0.3768 | 0.5436 |
| P5S | 4 | 345 | 0.5245 | 0.8055 | 0.5810 |
| P6S | 4 | 158 | 0.9262 | 0.1944 | 0.1402 |
| P1M | 3 | 59 | 0.8426 | 0.3326 | 0.3028 |
| P2M | 3 | 43 | 0.7144 | 0.5554 | 0.5056 |
| P3M | 3 | 359 | 0.6690 | 0.6028 | 0.5487 |
| P4M | 2 | 5 | 0.6800 | 0.5004 | 0.7219 |
| P5M | 3 | 50 | 0.8856 | 0.2652 | 0.2414 |
| P6M | 3 | 16 | 0.7734 | 0.4634 | 0.4218 |
| Teste t |  |  | -0.39 | 0.15 | -0.19 |
| Teste Mann-Whitney | 0.8 | 1.92 |  |  |  |
| p valor | 0.21 | 0.02* | 0.35 | 0.44 | 0.42 |

Collection point: trap in the ground (P1S, P2S, P3S, P4S, P5S, and P6S) and trap at the height of 3m (P1M, P2M, P3M, P4M, P5M, and P6M).
