# Supplementary material for: Spawning behavior of Aedini (Diptera: Culicidae) in a remnant of Atlantic Forest in the state of Rio de Janeiro
Source: Parasit Vectors. 2021 Nov 27;14:591. doi: 10.1186/s13071-021-05102-9 (PMC8626988; doi:10.1186/s13071-021-05102-9)
Supplement: Supplementary file 3 — Additional file 3: Table S3. Values of the co-occurrence C-score indices between each pair of mosquitoes species found. [file 13071_2021_5102_MOESM3_ESM.doc]

**Table S3** Values of the co-occurence C-score índices between each pair of mosquitoes species found.

| Species | *Ae. albopictus* | *Ae. terrens* | *Hg. janthinomys* | *Hg. leucocelaenus* |
| --- | --- | --- | --- | --- |
| *Ae. albopictus* |  | 35.00000 | 168.00000 | 0.00000 |
| *Ae. terrens* |  |  | 28.00000 | 30.00000 |
| *Hg. janthinomys* |  |  |  | 104.00000 |
